# Laparoscopic surgery for female posterior urethral bladder diverticulum with bladder outlet obstruction: A case report

**DOI:** 10.1097/MD.0000000000034971

**Published:** 2023-09-01

**Authors:** Chongzhou Liao, Zhenqiang He, Xiaoxiao Wang, Pu Guo, Wei Xiong

**Affiliations:** a School of Medicine, University of Electronic Science and Technology of China, Chengdu, China; b Department of Urology, Sichuan Academy of Medical Sciences and Sichuan Provincial People’s Hospital, Chengdu, China; c School of Medical and Life Sciences, Chengdu University of Traditional Chinese Medicine, Chengdu, China.

**Keywords:** bladder diverticula, case report, cystoplasty, female bladder outlet obstruction, laparoscopic diverticulectomy

## Abstract

**Introduction:**

Bladder diverticula (BD) can be classified into congenital and acquired forms, with the latter accounting for approximately 90% of all cases, primarily among male patients. Although BD-associated anatomical bladder outlet obstruction (BOO) is uncommon, existing literature suggests that congenital BD are more frequently observed in male children and rarely in female children. While around 70% of acquired BD are linked to BOO secondary to benign prostatic hyperplasia in male patients, clinical reports of female BD are less common. Furthermore, cases of female BD located posterior to the urethra, which lead to voiding difficulties, are exceedingly rare.

**Case presentation:**

Herein, we present a case of laparoscopic treatment in a 53-year-old female patient diagnosed with congenital bladder diverticulum causing progressively worsening dysuria. Voiding cystourethrography revealed a soft cystic protrusion of the posterior urethral wall during voiding, which reinforced the patient’s symptoms. Urodynamic examination showed elevated detrusor muscle contraction during voiding, a reduced urinary flow rate, and P/Q values indicative of significant BOO. Considering the patient’s clinical condition, we performed laparoscopic bladder diverticulectomy, partial urethral croppingplasty, and cystoplasty.

**Results:**

The laparoscopic bladder diverticulectomy, partial urethral croppingplasty, and cystoplasty procedures were completed thoroughly and with great success. However, complete removal of the diverticular epithelium proved challenging, resulting in an overall operative time of approximately 3 hours and 32 minutes. At the postoperative follow-up, the patient presented with symptoms of a lower urinary tract infection for a week, which were effectively resolved with oral antibiotics. At the 8-month follow-up, the patient reported normal urination and the absence of any discomfort during urination.

**Conclusion:**

Female bladder outlet obstruction resulting from posterior urethral BD can be challenging to visualize during transurethral cystoscopy, especially with limited angulation, and may even be overlooked. Furthermore, conventional transvaginal diverticulectomy is often difficult to perform effectively. Therefore, laparoscopic bladder diverticulectomy, partial urethral croppingplasty, and cystoplasty are considered appropriate treatment options for such cases.

## 1. Introduction

Bladder diverticula (BD) are mucosal protrusions that occur due to a defect in the detrusor muscle and consist of uroepithelium without a muscular layer, resulting in a thin diverticular wall.^[1]^ This can lead to urine accumulation, voiding difficulties, incontinence, urolithiasis, infection, and even diverticular cancer. Most patients present with these symptoms, which prompt a diagnosis.^[2]^ Acquired BD are commonly associated with bladder outlet obstruction (BOO), as are benign prostatic hyperplasia and recurrent infections.^[3]^ Currently, little is known about the diagnosis and treatment of BD, and reports on the efficacy of treating diverticula combined with BOO are limited,^[4]^ with posterior BD being even rarer. Over the past few years, surgical treatment of BD has been attempted using various approaches, including open, endoscopic, and laparoscopic techniques.^[5]^ Additionally, cases of diverticulectomy with the assistance of the da Vinci robot have also been reported.^[6]^ This report describes a case of a bladder diverticulum in a unique location, for which we performed cystectomy, partial urethral croppingplasty, and cystoplasty, resulting in a favorable prognosis.

## 2. Case presentation

**Figure 1.**
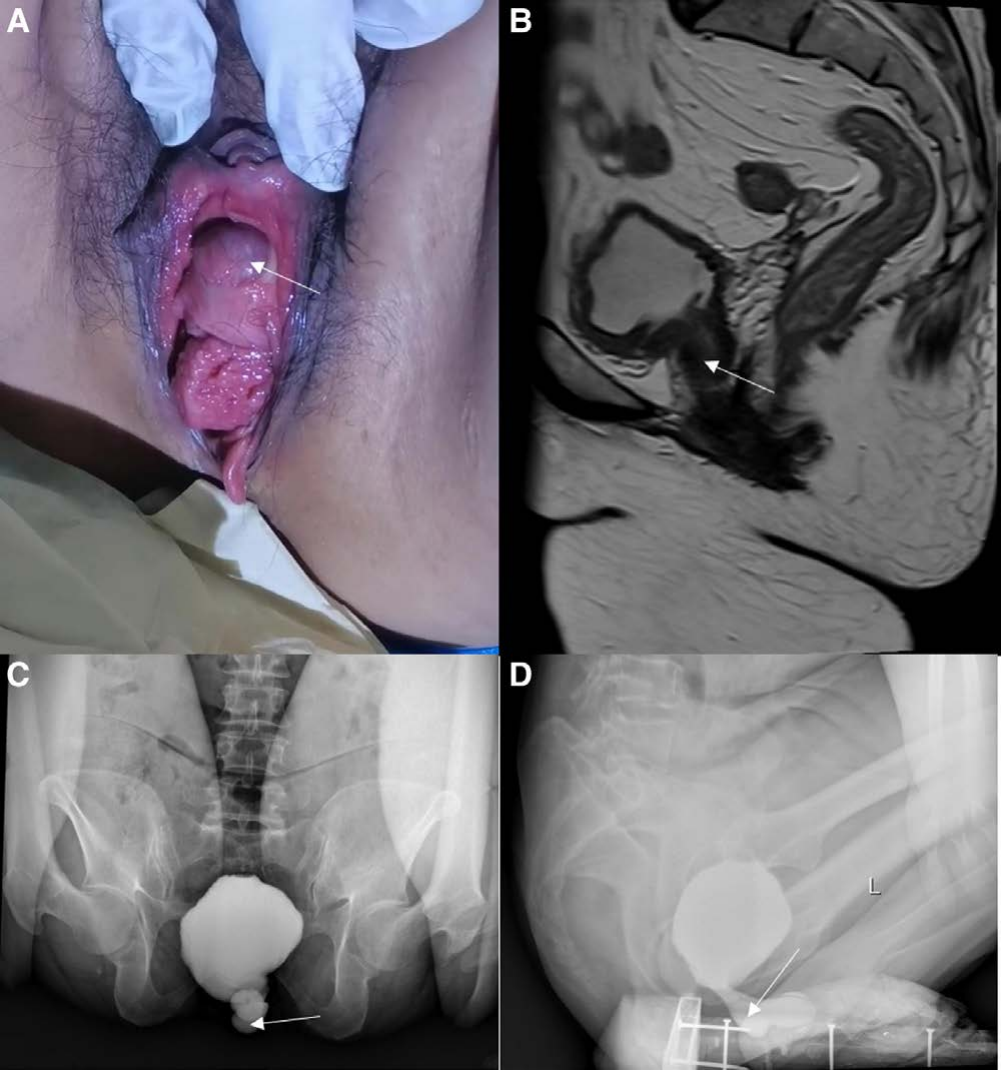
(A). Cystic protrusion of the posterior urethral wall. (B–D) The radiological manifestations of bladder diverticulum on MRI and VCUG. VCUG = voiding cystourethrography.

**Figure 2.**
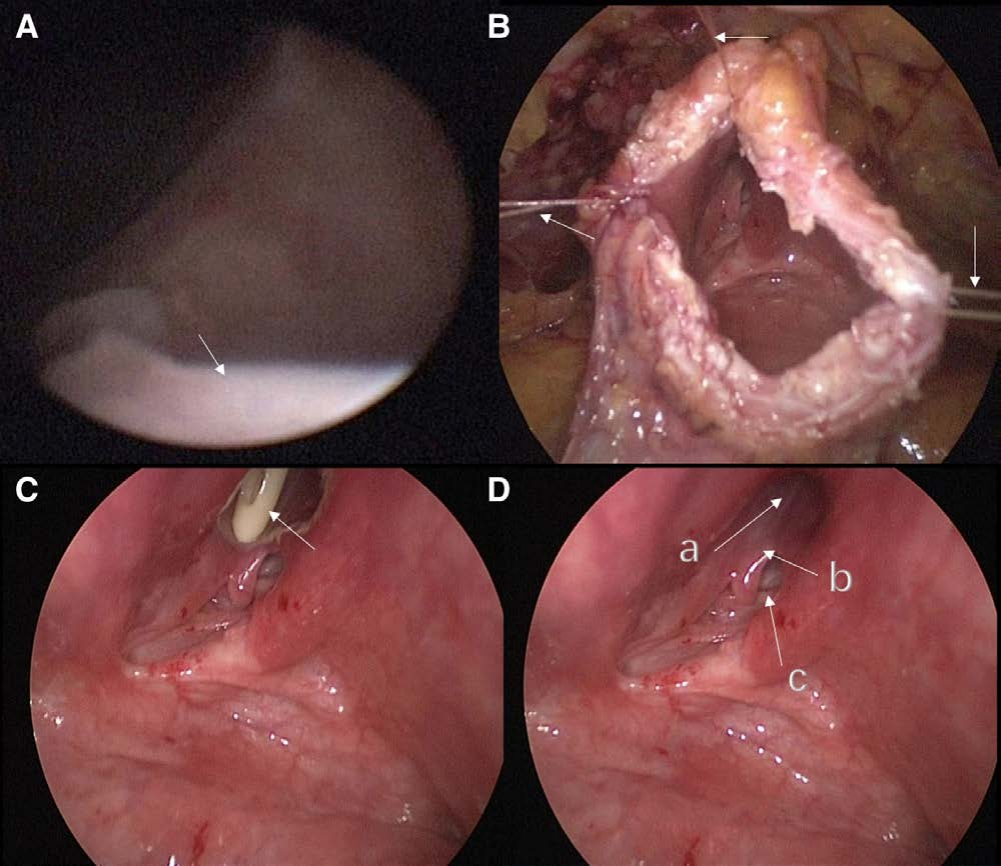
(A) The diverticulum wall observed during cystoscopy. (B) The bladder wall suspended by sutures ensures an excellent surgical field of view. (C, D) Under the guidance of a Foley catheter, clear visualization of the following structures: (a) Internal urethral orifice, (b) posterior wall of the urethra, which is also the anterior wall of the diverticulum, (c) neck of the diverticulum.

The patient, a 53-year-old woman, had a history of intermittent mild voiding difficulties and lower urinary tract symptoms (LUTS) for many years. She previously underwent left nephrectomy, left ureter, and partial cystectomy due to left renal atrophy. Two months after the operation, she experienced progressive worsening voiding difficulties. During urination, a cystic bulge measuring approximately 1.0 × 1.0 cm was observed in the posterior wall of the urethra (Fig. 1A). Pelvic MRI and voiding cystourethrography (VCUG) revealed a large cystic mass located in the posterior part of the urethra, which increased in size with the rise in abdominal pressure during voiding (Fig. 1B–D). Urodynamic examination demonstrated increased detrusor muscle contraction during voiding, decreased urinary flow rate, and significant bladder outlet obstruction indicated by P/Q values. Due to difficulties in urinary discharge, the free flow rate could not be measured. Based on the patient’s medical history and clinical data, we diagnosed her with a congenital posterior urethral bladder diverticulum. The location of the bladder diverticulum was unique, as the posterior wall of the urethra served as the anterior wall of the diverticulum, forming this distinct bladder diverticulum. The patient’s previous surgical history may have altered the normal bladder anatomy, leading to the enlargement of the diverticulum. This, in turn, compressed the bladder neck opening, resulting in BOO. The obstructive factors further contributed to the enlargement of the diverticulum, creating a vicious cycle that exacerbated the symptoms. Preoperative cystoscopy revealed significant trabecular growth of the bladder and an exposed diverticular wall, although complete visualization of the diverticulum was hindered by anatomical limitations (Fig. 2A). After reviewing the relevant literature and considering the patient’s financial situation, we decided to proceed with a purely laparoscopic approach for bladder diverticulectomy, urethral cropping, and cystoplasty.

**Figure 3.**
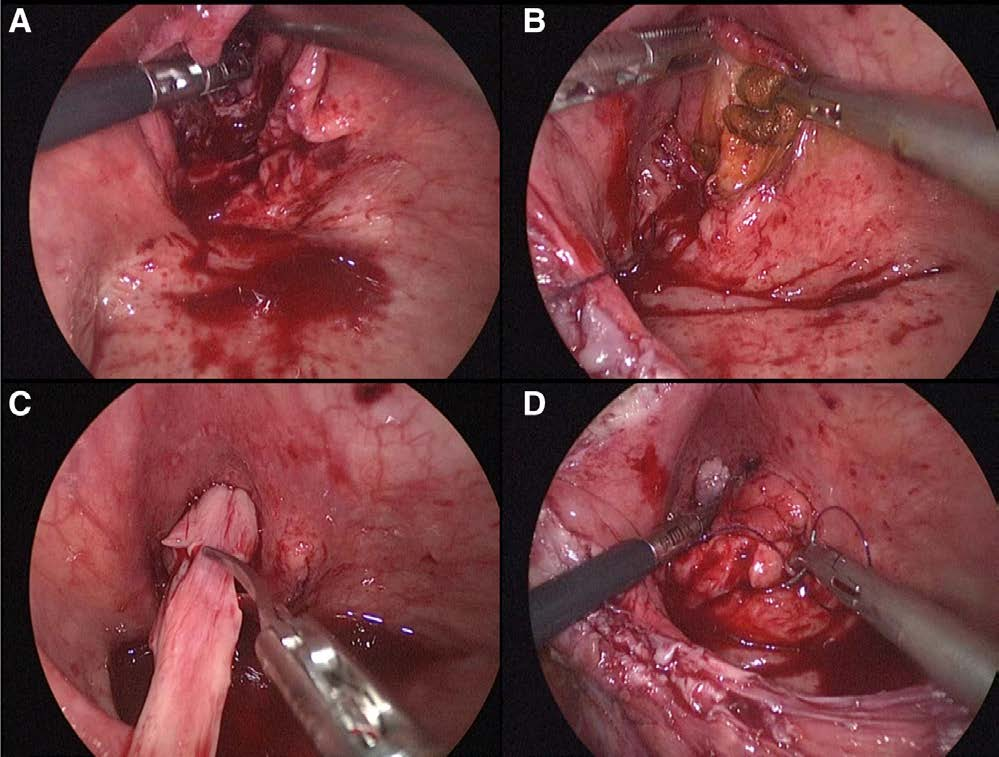
The inner wall epithelium of the diverticulum was meticulously excised, ensuring efficacy with iodine-soaked cotton balls. The diverticulum underwent trimming and shaping, followed by closure.

**Figure 4.**
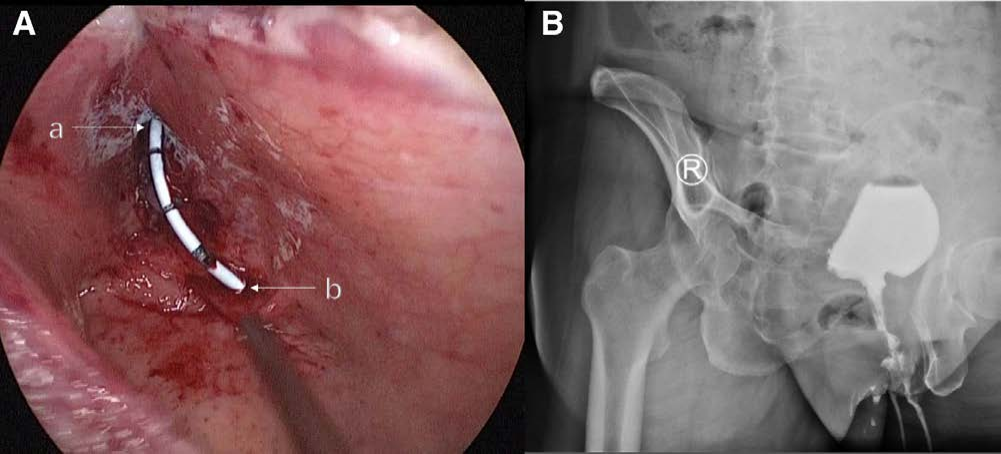
(A)(a) Internal urethral orifice, (b) ureteral orifice. (B) The VCUG obtained one month after surgery. VCUG = voiding cystourethrography.

The surgical approach was approved by the patient and their family, and informed consent was obtained. Under general anesthesia, the patient was positioned in the lithotomy position. Cystoscopy confirmed the previous findings. After removing the cystoscope, the surgical area was sterilized, and a trocar was inserted to establish pneumoperitoneum and gain access to the surgical site. The bladder was laparoscopically incised layer by layer, and sutures were used to suspend the bladder wall, ensuring a clear surgical view (Fig. 2B). With the guidance of a Foley catheter, we were able to visualize the anatomical relationship between the internal urethral opening, the diverticular opening, and the bladder neck. It is noteworthy that the posterior wall of the urethra, in its free state, contributed to the formation of part of the bladder diverticulum (Fig. 2C and D). Initially, we meticulously peeled the diverticular epithelium (Fig. 3A). This step took considerable time due to the tight adherence of the diverticular epithelial tissue to the subcutaneous tissue, and the absence of a natural anatomical gap that would aid in the peeling process. Iodine was then applied to enhance de-epithelialization (Fig. 3B). Next, excess free tissue from the posterior wall of the urethra was excised and shaped (Fig. 3C). The diverticular lumen was completely closed using a barbed suture (Fig. 3D). Subsequently, bladder neck plasty was performed, and a ureteral stent was inserted to divert urine from the ureters to the external urethral opening (Fig. 4A). This measure facilitates incision healing and reduces surgical complications. Finally, the bladder and abdominal wall incisions were closed layer by layer. The total operative time was 3 hours and 32 minutes, with an estimated blood loss of 60 cc.

## 3. Results

On the 14th day after surgery, the patient’s catheter and ureteral stent were removed. Her urination returned to normal, and the cystic protrusion at the urethral orifice disappeared. At the 1-month postoperative follow-up, a VCUG revealed the absence of the diverticulum and the restoration of urination function (Fig. 4B). The postoperative pathology report indicated chronic active inflammation and adenoid cystitis changes in the bladder mucosa. The diverticular wall of the bladder neck and partial urethra showed chronic active inflammation with squamous epithelial and glandular epithelial metaplasia. No evidence of malignancy was found. At the 8-month postoperative follow-up, the patient did not experience any LUTS symptoms or urinary abnormalities. Both the patient and her family expressed satisfaction with the treatment outcome and confidence in her future quality of life.

## 4. Discussion

Female bladder outlet obstruction is relatively uncommon compared to its male counterpart. Female bladder outlet obstruction caused by BD is even rarer, and the unique nature of this patient’s posterior urethral bladder diverticulum requires a specific surgical approach. The prevalence of congenital diverticula is approximately 1.7%, with an increasing incidence associated with age and a higher prevalence in men with benign prostatic hypertrophy, reaching up to 6%. This condition exhibits a 9:1 male-to-female ratio.^[7]^ Studies from the early 20th century have emphasized the importance of addressing the bladder neck outlet during diverticulectomy to prevent recurrence due to elevated voiding pressure. This principle remains one of the fundamental aspects of contemporary surgical management of BD.^[8]^ The term “Hatch diverticula” was coined by Hatch in the 1950s to describe BD associated with the ureteral orifice.^[9]^ On the other hand, primary or congenital diverticula occur in bladders with smooth inner walls, can be isolated or multiple, and develop without bladder outlet obstruction, distinguishing them from the classic “Hatch diverticula.”^[10]^ Acquired diverticula, on the other hand, are often associated with trabecular structures within the bladder, typically seen in cases of neurogenic bladder, posterior urethral valves, or bladder outlet obstruction due to severe voiding dysfunction.^[11]^ They may also occur as a result of medical conditions or after surgical procedures that weaken the detrusor muscle. Additionally, BD have been linked to genetic disorders such as Ehlers-Danlos syndrome and Williams-Beuren syndrome.^[12]^ In our review, we also found evidence suggesting that bladder ultrasound may be more effective than VCUG in diagnosing BD in certain cases, particularly when anterior or posterior diverticula may not be clearly visualized during VCUG due to contrast obscuring the diverticula.^[13]^ This finding holds potential value for future pre-diagnostic evaluations of such diverticula.

In this particular case, we opted against the classic transvaginal diverticulectomy due to limitations in the operating space and the likelihood of increased operative time. Additionally, the specific anatomical location of the bladder diverticulum would have resulted in a lower success rate for this type of surgery. Importantly, postoperative complications such as recurrent diverticula, LUTS, recurrent urinary tract infections, de novo stress urinary incontinence, and urethrovaginal fistula are challenging complications that can negatively impact the prognosis of the procedure. Therefore, we chose the laparoscopic procedure as a more reliable and straightforward approach.^[14]^ During cystoscopy before the radical left nephrectomy, a “ladder-like” local structure of the diverticulum was observed. However, due to the direction of the cystoscope’s opening, it was challenging to visualize the entire diverticulum. It was not until the bladder was opened laparoscopically that we fully understood the overall location and structure of the diverticulum. The diverticulum was formed by the posterior wall of the urethra and the bladder wall, with the posterior wall of the urethra extending into the bladder cavity as a free structure, resembling a “live flap.” During urination, increased bladder muscle tension led to elevated bladder pressure, causing the diverticulum to fill up. The side wall of the diverticulum (posterior urethral wall) would press against the urethral orifice, resulting in mechanical difficulty in urination during voiding. A similar case has been reported in a single case report, although the surgical approach was not described in detail.^[15]^

In this surgery, we completely removed the epithelial-like structures in the diverticular wall to minimize the risk of recurrent formation of secondary cystic cavities. The excessive tissue of the posterior wall of the urethra was also appropriately addressed to alleviate the obstructive effect. By performing these surgical steps, we aimed to achieve a successful and sustainable outcome for the patient.

## Author contributions

**Conceptualization:** Xiaoxiao Wang.

**Investigation:** Xiaoxiao Wang, Pu Guo.

**Methodology:** Zhenqiang He.

**Writing – original draft:** Chongzhou Liao.

**Writing – review & editing:** Wei Xiong.
